# Interstitial Nephritis in a Patient with Inflammatory Bowel Disease

**DOI:** 10.1155/2016/4260365

**Published:** 2016-09-14

**Authors:** Payaswini Vasanth, Michelle Parmley, Jose Torrealba, Tamim Hamdi

**Affiliations:** ^1^Department of Internal Medicine, Division of Nephrology, UT Southwestern Medical Center, Dallas, TX, USA; ^2^Department of Pathology, UT Southwestern Medical Center, Dallas, TX, USA

## Abstract

Tubulointerstitial nephritis in patients with inflammatory bowel disease has been linked to the use of 5-ASA derivatives. Various aspects of this theory have been challenged with a potential role for the underlying autoimmune disorder. Steroids are the mainstay of treatment and mycophenolate mofetil might be an effective alternative. We report a patient who responded well to mycophenolate despite continuing mesalamine, the suspected offending agent.

## 1. Introduction

Tubulointerstitial nephritis (TIN) is a common cause of kidney injury [1] characterized by inflammatory interstitial infiltration leading to renal tubular damage and possibly irreversible scarring [2] which may result in end-stage renal disease [3]. 5-Aminosalicylic acid (5-ASA) commonly used to treat inflammatory bowel disease (IBD) is a well-reported cause of drug-induced AIN [4]. Renal involvement in patients with IBD is complex and not exclusively related to medication side effects, as it can represent an extraintestinal manifestation of the underlying immune pathophysiology or develop as a complication of those systemic groups of diseases [5].

## 2. Case Report

**Figure 1:**
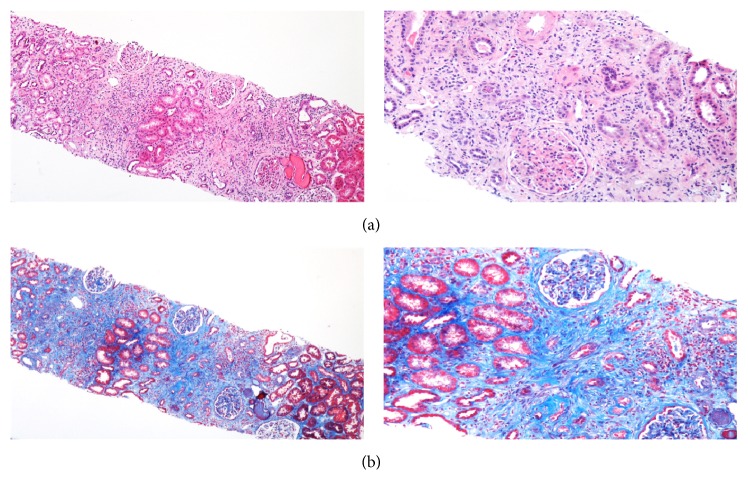
Light microscopic examination of the kidney biopsy showing severe cortical interstitial fibrosis and tubular atrophy, acute interstitial nephritis with prominent mononuclear cellular infiltrate, and multifocal tubulitis ((a) H&E 4x, 10x; (b) Masson Trichrome 4x, 10x).

A 65-year-old Caucasian female with past medical history of ulcerative colitis (UC) was referred to the nephrology clinic from an outside gastroenterology practice for evaluation of an elevated plasma creatinine level (P_Cr_) of 2.31 mg/dL from a baseline of 0.9 mg/dL 6 months earlier. She was diagnosed with UC 30 years ago and her most recent colonoscopy 2 months prior to presentation showed normal colonic mucosa but diffuse chronic active granulomatous colitis on pathologic examination suggestive of Crohn's-related process. She was asymptomatic and maintained on oral mesalamine. Laboratory workup was noted for sterile pyuria and a urine protein-to-creatinine ratio (UPCR) of 1 g/g but was otherwise unremarkable. Peripheral eosinophilia was absent. A renal ultrasound showed bilateral parenchymal echogenicity and a kidney biopsy revealed chronic active interstitial nephritis with infiltration by mononuclear cells and rare eosinophils, focal tubular microabscesses and tubular atrophy, effacement of epithelial foot processes, and severe fibrosis affecting 70% of the interstitium. Granulomas were absent (Figure 1). Immunofluorescence showed positive linear staining of the tubular basement membrane by C3 and the tubules by IgG.

**Figure 2:**
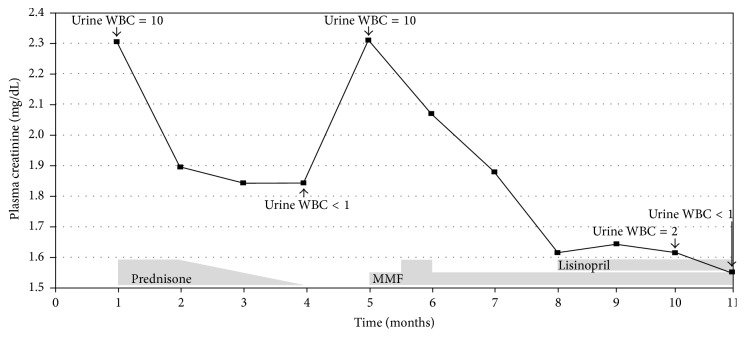
Changes in renal function over time. MMF: mycophenolate mofetil; WBC: white blood cells per high power field on microscopic examination of urine sediment.

Mesalamine was suspected to be the offending agent but was not discontinued per the recommendation of the referring gastroenterologist due to financial factors and the relative stability of her previously difficult-to-control disease treated with multiple agents in the past. The patient was started on prednisone in an attempt to salvage any remaining kidney function. She received 1 mg/kg daily for four weeks followed by a taper over 8 weeks. By the end of this course, P_Cr_ improved to 1.84 mg/dL and the pyuria disappeared. One month later, P_Cr_ worsened again to 2.32 mg/dL and the pyuria recurred. Given the relapse shortly after prednisone taper and a recently diagnosed osteoporosis, we opted to attempt treatment with mycophenolate mofetil (MMF). The dose was uptitrated to 1 g twice a day resulting in improvement of P_Cr_ to 1.88 mg/dL. The dose was later reduced to 500 mg twice a day due to gastrointestinal side effects and P_Cr_ continued to improve and reached 1.54 mg/dL. The UPCR stabilized at 0.3 g/g while the pyuria disappeared. Lisinopril was started (Figure 2). A repeat colonoscopy while the patient was receiving MMF showed again a normal mucosa but this time minimally active chronic colitis with resolution of granulomas on pathologic examination improved compared to the prior findings.

## 3. Discussion

The spectrum of renal involvement in patients with IBD is broad and includes nephrolithiasis, glomerulonephritis (commonly IgA nephropathy), tubulointerstitial nephritis (TIN), and renal amyloidosis [5, 6]. TIN is particularly associated with the use of 5-ASA [4]. This has been shown in a review of 46 reported cases where TIN was biopsy-proven in 42 cases, and withdrawal of 5-ASA resulted in variable improvement in renal function thus suggesting a cause-effect relationship [4]. A retrospective analysis of 171 patients receiving 5-ASA over a mean duration of 8.4 years challenged those findings and reported a subtle dose and duration-dependent decline in renal function, with only one case being attributed to TIN [7]. However, kidney biopsies were not routinely performed to exclude TIN. Other studies suggested that TIN in patients with IBD is not exclusively related to 5-ASA use and described a disease progression similar to the usual extraintestinal manifestations associated with IBD, thus hypothesizing an underlying autoimmune etiology. TIN has been biopsy-proven in patients with IBD and absent 5-ASA exposure [6, 8, 9] and progressed sometimes independent of the intestinal disease activity [9, 10]. This makes the identification of the underlying etiology of TIN in patient with IBD receiving 5-ASA challenging, as TIN can develop through an independent autoimmune mechanism even if the intestinal disease is inactive and gets falsely attributed to 5-ASA use. From a histologic perspective, the presence of granulomas with or without eosinophils on kidney biopsy suggests a drug reaction, but this finding is of limited sensitivity. In a recent series of sixteen cases of biopsy-proven TIN, nine patients had known exposure to 5-ASA, of which 5 had granulomatous TIN. None of the remaining 7 cases with no exposure to 5-ASA had granulomatous TIN indicating that this specific pathologic finding occurred in about half of the cases exposed to 5-ASA but exclusively in this group [6]. The presence of immunofluorescence staining of the tubules and tubular basement membrane favors an autoimmune process although this pattern can be seen with other conditions and with drugs, particularly but not exclusively with antibiotics.

In the presented case, it is unclear whether TIN was a drug reaction or an extraintestinal manifestation of IBD, especially with simultaneous appearance of granulomas in the colon suggesting newly diagnosed Crohn's disease. Similarly, the response to MMF could have been due to a direct effect on the kidney or secondary to an improvement in the colitis as noted on subsequent biopsies, where colonic granulomas disappeared and the disease activity became minimal. MMF is not a recommended treatment for IBD but multiple studies suggested its possible benefits [11, 12].

Besides discontinuation of the offending agent, corticosteroids are the best available treatment for TIN especially when administered early after diagnosis [13]. Steroid-dependence, resistance, or contraindication can be managed with MMF. The optimal data comes from Preddie et al. where 8 patients with steroid-resistant and biopsy-proven acute interstitial nephritis received MMF at 1-2 g/day for a mean of 24.3 months leading to a mean decline in P_Cr_ from 2.3 to 1.6 mg/dL. Five patients successfully discontinued MMF after a mean follow-up of 28 months [14]. Our patient received 1 g/day of MMF and had a comparable improvement in P_Cr_ despite continuation of mesalamine, the possible offending agent. If mesalamine was indeed the offending agent, the clinical improvement our patient had with the simultaneous use of MMF is intriguing.

## References

[1] López-Gómez J. M., Rivera F., Spanish Registry of Glomerulonephritis (2008). Renal biopsy findings in acute renal failure in the cohort of patients in the Spanish registry of glomerulonephritis. *Clinical Journal of the American Society of Nephrology*.

[2] Praga M., González E. (2010). Acute interstitial nephritis. *Kidney International*.

[3] Clarkson M. R., Giblin L., O'Connell F. P. (2004). Acute interstitial nephritis: clinical features and response to corticosteroid therapy. *Nephrology Dialysis Transplantation*.

[4] Gisbert J. P., González-Lama Y., Maté J. (2007). 5-Aminosalicylates and renal function in inflammatory bowel disease: a systematic review. *Inflammatory Bowel Diseases*.

[5] Corica D., Romano C. (2016). Renal involvement in inflammatory bowel diseases. *Journal of Crohn's & Colitis*.

[6] Ambruzs J. M., Walker P. D., Larsen C. P. (2014). The histopathologic spectrum of kidney biopsies in patients with inflammatory bowel disease. *Clinical Journal of the American Society of Nephrology*.

[7] Patel H., Barr A., Jeejeebhoy K. N. (2009). Renal effects of long-term treatment with 5-aminosalicylic acid. *Canadian Journal of Gastroenterology*.

[8] Izzedine H., Simon J., Piette A. M. (2002). Primary chronic interstitial nephritis in Crohn's disease. *Gastroenterology*.

[9] Marcus S. B., Brown J. B., Melin-Aldana H., Strople J. A. (2008). Tubulointerstitial nephritis: an extraintestinal manifestation of Crohn disease in children.. *Journal of pediatric gastroenterology and nutrition*.

[10] Waters A. M., Zachos M., Herzenberg A. M., Harvey E., Rosenblum N. D. (2008). Tubulointerstitial nephritis as an extraintestinal manifestation of Crohn's disease. *Nature Clinical Practice Nephrology*.

[11] Smith M. R., Cooper S. C. (2014). Mycophenolate mofetil therapy in the management of inflammatory bowel disease—a retrospective case series and review. *Journal of Crohn's & Colitis*.

[12] Renna S., Cottone M., Orlando A. (2014). Optimization of the treatment with immunosuppressants and biologics in inflammatory bowel disease. *World Journal of Gastroenterology*.

[13] González E., Gutiérrez E., Galeano C. (2008). Early steroid treatment improves the recovery of renal function in patients with drug-induced acute interstitial nephritis. *Kidney International*.

[14] Preddie D. C., Markowitz G. S., Radhakrishnan J. (2006). Mycophenolate mofetil for the treatment of interstitial nephritis. *Clinical Journal of the American Society of Nephrology*.

